# Percutaneous Ureteral Stent Insertion in Patients with Benign Uretero-Ileal Anastomosis Strictures Who Underwent Radical Cystectomy: Assessing Risk Factors for Stent Patency

**DOI:** 10.3390/jcm12247721

**Published:** 2023-12-16

**Authors:** Yebin Kim, Chang Hoon Oh, Sang Lim Choi, Sungwon Kim

**Affiliations:** 1 Department of Radiology, Ewha Womans University Mokdong Hospital, College of Medicine, Ewha Womans University, Seoul 07985, Republic of Korea; 21853@eumc.ac.kr; 2 Department of Radiology, Chung-Ang University Gwangmyeong Hospital, Gwangmyeong 14353, Republic of Korea; sanglim84@cauhs.or.kr; 3 Department of Radiology, Research Institute of Radiological Science, Yongin Severance Hospital, Yonsei University College of Medicine, Yongin 03186, Republic of Korea; kswpig@yuhs.ac

**Keywords:** cystectomy, ureteral stent, bladder cancer, stent dysfunction, urinary

## Abstract

We aimed to investigate the risk factors of early double-J ureteral stent (DJUS) dysfunction rates and the long-term patency of DJUSs inserted via a percutaneous approach in patients with benign uretero-ileal anastomosis stricture (UIAS) who had undergone radical cystectomy. In this retrospective study, 63 DJUS placements were placed via a percutaneous nephrostomy tract in 42 consecutive patients between May 2020 and March 2023. The technical success rate was 100% in all patients without major complications. The early dysfunction rate and long-term patency rate were 38.1% (24/63) and 84.2% (32/38), respectively. The blood clot retention grade, balloon dilatation, and length of the ureteral stricture exhibited a significant correlation with early DJUS dysfunction (blood clot retention grade: odds ratio (OR) 6.922 in grade two, *p* = 0.009; balloon dilatation: OR 0.186, *p* = 0.017; length of ureteral stricture: OR 8.715, *p* = 0.035 in moderate stenosis, and 7.646, *p* = 0.028 in severe stenosis). A multivariate Cox’s proportional hazard analysis revealed that blood clot retention grade and length of ureteral stricture were independent predictors of long-term DJUS patency. Percutaneous insertion of the DJUSs was safe and effective in patients with benign UIAS.

## 1. Introduction

Radical cystectomy is indicated for surgical candidate patients with muscle-invasive or high-risk non-muscle-invasive bladder cancer and who are willing to undergo urinary diversion. While several options are available for urinary tract reconstruction, the incontinent ileal conduit and continent orthotopic neobladder are the most utilized urinary diversions [1,2].

Although the incidence of post-surgical double-J ureteral stent (DJUS) insertion has decreased, the potential issue of benign ureteroileal anastomotic stricture (UIAS) remains and can occur in 0–19% of patients within 1–2 years of performing urinary diversion [3,4]. UIAS can occur due to ureteroenteric ischemia resulting from the extended separation of the distal end of the ureter and poor suturing techniques, leading to fibrous scar hyperplasia [5]. Should the ureteral outlet stricture not be addressed in a timely manner, complicated upper urinary tract infections, stones, renal insufficiency, and other serious complications may develop [6,7,8]. DJUS insertion is the most used method for the treatment of benign ureteral strictures. Ureteral stents can be used alone or in combination with balloon dilation [9,10]. Previous studies have shown that DJUSs after balloon dilation can prevent ureteral rebound and scar contraction, help drain urine, and restore renal function.

Although ureteroscopy is typically performed retrogradely to the ureter, this approach cannot be completed in patients with low ureteral strictures because of a narrowed ureteral opening or failure of the guidewire passage [11,12]. In a few cases, retrograde identification of the ureteral orifice or anastomosis site can be difficult because of anastomotic tortuosity in patients with UIAS who have undergone radical cystectomy and urinary diversion. Therefore, the antegrade approach for DJUS insertion is technically easier and more effective.

Bladder cancer is the ninth-most common cancer worldwide, and its incidence is increasing annually [13]. However, there is a lack of research and understanding of the patency and efficacy of DJUSs in treating benign UIAS that occurs post-radical cystectomy. Therefore, in this study, we investigated the risk factors for early DJUS dysfunction and the long-term patency of DJUSs inserted percutaneously in patients with benign UIAS who had undergone radical cystectomy. 

## 2. Materials and Methods

### 2.1. Patient Population

This retrospective, single-center study was approved by the institutional review board, and thus the need for informed consent from the patients was waived (EUMC-2023-09-009). All patients who underwent radical cystectomy because of bladder cancer with an ileal conduit or an ileal orthotopic neobladder for bladder cancer were included. Patients who underwent antegrade internal ureteral stent placement at our hospital between May 2020 and March 2023 were enrolled. Eighty antegrade DJUS procedures were performed via percutaneous nephrostomy (PCN) in 51 consecutive patients. The inclusion criteria were an age of 40–90 years, patients with benign UIAS undergoing radical cystectomy, and confirmation of overt hydronephrosis via computed tomography (CT). The exclusion criteria were an expected life expectancy of less than three months, ureteral stricture due to malignancy, and poor general health status (Eastern Cooperative Oncology Group performance status grade four). 

### 2.2. Procedure

**Figure 1:**
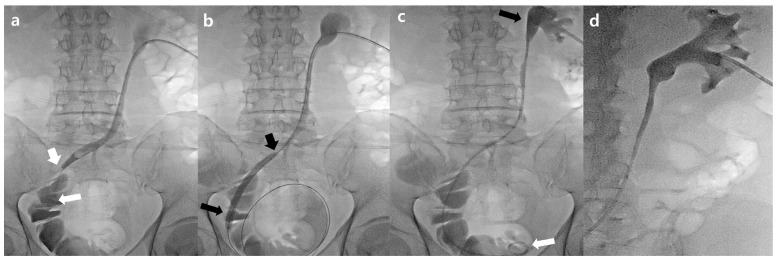
A 63-year-old man with a uretero-ileal anastomosis stricture (UIAS) who had undergone radical cystectomy and already had percutaneous nephrostomy (PCN). (**a**) After 8F sheath insertion via the previous PCN route, a nephrogram showed long segmental stenosis (2.4 cm) from the distal ureter to the uretero-ileal anastomosis site (arrow). (**b**) Balloon dilatation was performed using a 6 mm balloon catheter (arrow) at the stricture portion of the distal ureter. (**c**) After balloon dilatation, a 6F double-J ureteral stent insertion was performed, and the proximal tip located the renal pelvis (black arrow) and the distal tip located the orthotopic neobladder (white arrow). (**d**) Post procedure, a nephrogram showed no blood clots in one or more calyces (blood clot retention grade one).

Two days after PCN, antegrade nephrography was performed to evaluate the level and length of the obstruction. An 8F introducer sheath (Pinnacle TIF Tip, Terumo, Tokyo, Japan) was introduced over a 0.035 inch hydrophilic stiff guidewire (Radifocus, Terumo), and a 5F angiographic catheter was inserted over the guidewire to pass the UIAS site into the orthotopic neobladder or ileal conduit. Balloon dilatation was performed using a 4–6 mm balloon catheter (Mustang, Boston Scientific, Natick, MA, USA; or Genoss Co., Ltd., Suwon, Republic of Korea) in cases of significant stenosis or occlusion, which made it difficult for the guidewire or catheter to pass through. Finally, a 6 or 8F DJUS (Flexima, Boston Scientific, Natick, MA, USA; or Inlay Optima, BD, Tempe, AZ, USA) was inserted at the proximal end of the renal pelvis and the distal end within the orthotopic neobladder or ileal conduit. The Flexima ureteral stent had three side holes in the proximal 2 cm of the straight portion, whereas the InLay Optima ureteral stent had multiple side holes in the straight portion. In all patients with successful stent placement, a PCN tube was inserted to determine the patency of the stent, which was subsequently clamped when the drained urine was clear or mild hematuria was observed (Figure 1). 

Clamping of the PCN tube was performed one or two days after DJUS placement. During clamping, signs and symptoms suggestive of DJUS malfunction and recurrent hydronephrosis, including urine leakage via the PCN tract, flank pain, and fever, were evaluated. Finally, the PCN tube was removed two or three days after the procedure, given that the nephrogram confirmed good patency and satisfactory positions of the stents or if the patients did not demonstrate any noteworthy symptoms or signs, such as flank pain, fever, or urine leakage via the PCN tract. 

### 2.3. Study Endpoints

The patency of the internal ureteral stent was confirmed using renal biochemistry or imaging studies, including ultrasonography or CT, at 1 and 3 months post insertion. Early DJUS dysfunction was defined as no passage of contrast media into the urinary bladder on a post-procedural nephrogram performed using a PCN catheter 2–3 days after DJUS placement, or the occurrence of symptoms and signs suggesting DJUS malfunction during clamping of the PCN catheter before the nephrogram. Furthermore, we included cases of early DJUS dysfunction if ureteral obstruction or DJUS replacement was suspected within two weeks, regardless of the patency indication from the post-procedural nephrogram. Long-term patency was defined as the absence of functional abnormalities for 3–4 months (time point for the first routine DJUS change) in patients without early DJUS dysfunction. 

**Figure 2:**
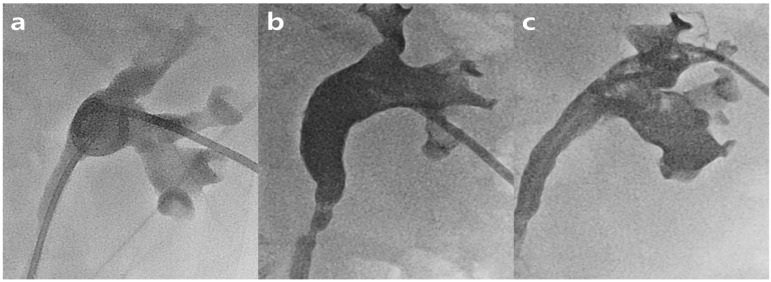
Blood clot retention grades. Grade 1 (**a**), retention of minimal or no blood clots in one or more calyces or the infundibulum alone; grade 2 (**b**), retention of blood clots in less than half of the renal pelvis; and grade 3 (**c**), retention of blood clots in most of the renal pelvis and/or ureter.

The blood clot retention grade was evaluated using blood clots in the renal pelvis on a three-point scale: grade 1, retention of minimal or no blood clots in one or more calyces or the infundibulum alone; grade 2, retention of blood clots in less than half of the renal pelvis; and grade 3, retention of blood clots in most of the renal pelvis and/or ureter (Figure 2). Hydronephrosis was graded according to the Onen grade system [14]. Ureteral stricture length was graded according to the following criteria: mild (<1 cm), moderate (1–2 cm), and severe (>2 cm). Technical success was defined as a successful stent placement at the desired location. Complications were classified as minor or major according to the Society of Interventional Radiology guidelines [15].

### 2.4. Follow-Up

All patients were clinically evaluated using renal biochemistry tests (e.g., blood urea nitrogen (BUN) or serum creatinine (Cr)), plain abdominal radiography, and abdominal CT scans three months post procedure. Patients presenting with unexpected symptoms such as urinary frequency, flank pain, dysuria, hematuria, or fever were urgently evaluated. Retrograde DJUS changes were evaluated under cystoscopy in cases where a residual stricture was suspected. If no significant clinical, laboratory, or imaging findings were observed after three months, the DJUS was removed. 

### 2.5. Statistical Analysis

Continuous variables are presented as their mean ± standard deviation values and were compared using the Student’s *t*-test. Categorical variables were compared using the chi-square or Fisher’s exact test. Univariate and multivariate logistic regression analyses of risk factors for early DJUS dysfunction were performed. Univariate Cox proportional hazards analyses of the risk factors associated with overall DJUS patency were performed. All statistical analyses were performed using SPSS software version 20.0. (SPSS Inc., Chicago, IL, USA), with *p*-values < 0.05, indicating statistical significance. 

## 3. Results

Patient characteristics are shown in Table 1. Sixty-three antegrade DJUS placements were performed in 42 patients with benign UIAS who had undergone radical cystectomy. The procedure was not performed in eight patients (sixteen cases) because of malignant ureteral stricture and one patient (one case) because of a uretero-jejunal fistula. There were 55 male and 8 female participants, with an average age of 68.8 ± 6.7 years. Among them, 59 had orthotopic neobladders, and 4 had ileal conduits. Hydronephrosis was graded according to the following criteria using the Onen grading system. Mild hydronephrosis: Onen grade one (15 cases, 23.8%), moderate hydronephrosis: Onen grade two (35 cases, 55.6%), and severe hydronephrosis: Onen grades three or four (13 cases, 20.6%). Blood clot retention grade was evaluated using a nephrogram immediately after DJUS placement. Of all placements, 38 (60.3%) had grade one blood clot retention, 18 (28.6%) had grade two blood clot retention, and 7 (11.1%) had grade three blood clot retention. The ureteral stricture was mild in 18 cases (28.6%), moderate in 15 cases (23.8%), and severe in 30 cases (47.6%). Balloon dilatation (4 mm (*n* = 6), 5 mm (*n* = 16), or 6 mm (*n* = 8)) was performed in 30 patients (47.6%) because of significant stenosis at the ureteroileal anastomosis. The preoperative BUN was 30.48 ± 15.27 mg/dL and preoperative Cr was 2.1 ± 1.4 mg/dL. 

**Figure 3:**
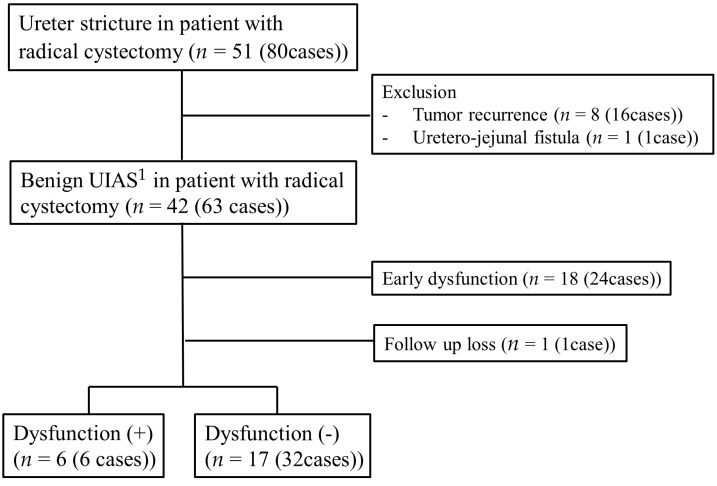
Flowchart of double-J ureteral stent procedures in patients with radical cystectomy and benign UIAS. ^1^ UIAS: uretero-ileal anastomosis stricture.

All 63 patients underwent the interventional operation via the antegrade approach, and the technical success rate was 100%, with no ureteral perforation, rupture, or other complications during the procedure. The early dysfunction and long-term patency rates were 38.1% (24/63) and 84.2% (32/38), respectively. One patient (one case) was excluded from the long-term DJUS patency evaluation due to loss to follow-up (Figure 3).

Univariate and multivariate logistic regression analyses were performed to identify risk factors associated with early DJUS dysfunction. In the multivariate analysis, blood clot retention grade, balloon dilatation before DJUS placement, and the length of ureteral stricture exhibited a significant association with early dysfunction of the DJUSs (odds ratio (OR) and confidence interval (CI) values for—blood clot retention grade: OR (95% CI) = 6.922 (1.611–29.744), *p* = 0.009 for grade two; balloon dilatation: OR (95% CI) = 0.186 (0.046–0.743), *p* = 0.017; and length of ureteral stricture: OR (95% CI) = 8.715 (1.164–65.226), *p* = 0.035 in moderate stenosis and 7.646 (1.245–46.953), *p* = 0.028 in severe stenosis) (Table 2). Early dysfunction occurred in 0% (0/8), 25% (2/8), and 35.7% (5/14) of patients who underwent balloon dilation for mild, moderate, and severe ureteral strictures, respectively. In patients who did not undergo balloon dilation, the rates were 20% (2/10), 71.4% (5/7), and 62.5% (10/16) for mild, moderate, and severe diseases, respectively. 

**Figure 4:**
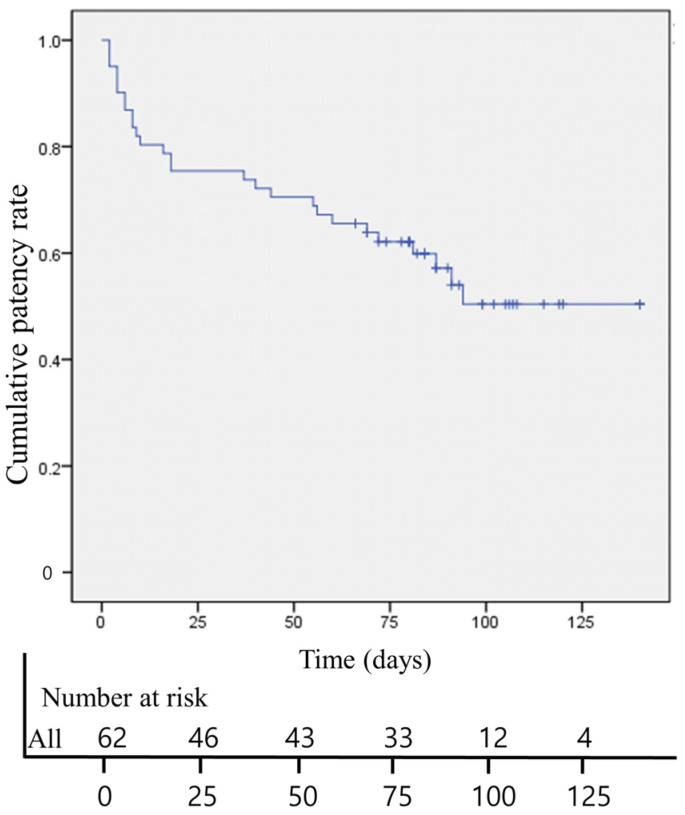
Kaplan–Meier analysis showing the overall double-J ureteral stent patency rate.

**Figure 5:**
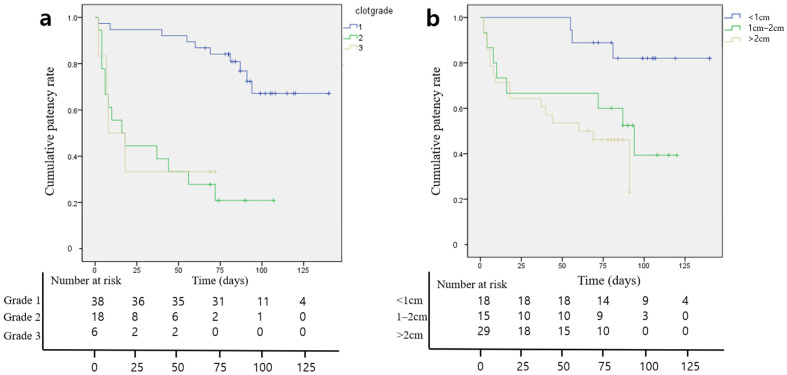
Kaplan–Meier analysis showing the overall double-J ureteral stent patency rate according to the blood clot retention grade (**a**) and length of the ureteral stricture (**b**). Overall stent patency rates were significantly decreased by increasing the blood clot grade (odds ratio (OR): 6.044, *p* < 0.001 for grade 2; 6.330, *p* = 0.004 for grade 3) and the length of ureteral stricture (OR: 4.081, *p* = 0.042 in moderate stenosis; 5.518, *p* = 0.015 in severe stenosis).

According to the Kaplan–Meier survival analysis, the median overall DJUS patency was 88 days (95% CI, 74–103 days) (Figure 4 and Figure 5). Hydronephrosis, blood clot retention grade, and ureteral stricture length were identified as factors for DJUS patency in the univariate Cox proportional hazards analysis. However, the multivariate Cox proportional hazards analysis revealed that the blood clot retention grade and length of ureteral stricture were independent predictors of long-term DJUS patency (blood retention grade: OR (95% CI) = 6.044 (2.463–14.831), *p* < 0.001 in grade two; OR (95% CI) = 6.330 (1.810–22.140), *p* = 0.004 in grade three; and length of ureteral stricture: OR (95% CI) = 4.081 (1.054–15.798), *p* = 0.042 in moderate stenosis, and 5.518 (1.399–21.764), *p* = 0.015 in severe stenosis) (Table 3). 

## 4. Discussion

This study revealed that early DJUS dysfunction occurred in 38.1% of patients with UIAS who had undergone radical cystectomy, which is consistent with the results of a previous study. DJUS dysfunction could be affected by balloon dilatation, increased ureteral stricture length, and blood clot retention grade, as determined in our multivariate logistic regression analysis [16]. Many previous studies have attempted to identify the risk factors for UIAS, and running anastomosis, postoperative urinary tract infection, intraoperative or postoperative blood transfusion, and extracorporeal bladder anastomosis have been found to increase the risk of UIAS; however, the precise cause remains unclear [17,18]. Many factors during surgery, such as the extent of management of the broken ureter end, degree of tissue dissociation, interruption of the ureteral blood supply, and tension after ureteral replantation, cannot be accurately measured or quantified [17]. Although surgical repair has a high success rate and prolonged patency, advancements in interventional radiologic techniques have resulted in the introduction of alternative treatments such as balloon dilation and ureteral stent placement [19]. 

Previous studies have confirmed that balloon dilatation has a significant effect on ureteral stenosis [20,21,22,23]. Li et al. reported 78 cases of lower ureteral stenosis when PCN was combined with balloon dilation for the treatment, with an effective rate of 92% and a technical success rate of 100% [24]. Yam et al. reported that balloon dilatation was safe and effective for the treatment of ureteral strictures, and they recorded DJUS patency rates at 3, 6, and 12 months of 81.4% (35/43), 86.2% (25/29), and 85% (17/20), respectively, indicating its long-term effectiveness [25]. Furthermore, DJUS placement after balloon dilatation can prevent ureteral rebound and scar contraction, facilitate urine drainage, and restore renal function [10]. In this study, balloon dilatation significantly reduced the incidence of early DJUS dysfunction (OR: 0.186; *p* = 0.017). However, the overall long-term patency of the DJUSs was not statistically significant (*p* = 0.781). This result might be consistent with those from previous studies that reported a high restenosis rate and short reintervention interval in patients with UIAS who underwent radical cystectomy [26,27]. Moreover, a previous study showed that balloon dilation of such strictures, even with a presumed intact vascular supply, had a 2-year patency rate of only 40% in patients with UIAS [28].

Youn et al. reported that early DJUS dysfunction rates significantly increased with higher blood clot retention grades (grade one: 28.9%, grade two: 57.6%, and grade three: 69.2%; *p* < 0.001). However, long-term patency was not significantly influenced by the blood clot retention grade (*p* = 0.949) [16]. In our study, the blood clot retention grade appeared to be a significant risk factor for early DJUS dysfunction (OR: 6.922, *p* = 0.009 in grade two). However, contrary to previous studies, as the blood clot retention grade increased, the risk of long-term patency also increased. Human urine is a primary source of urokinase, a fibrinolytic enzyme that resolves blood clots over time; therefore, most DJUS obstructions caused by blood clots are resolved by the first or second follow-up nephrogram [29]. However, not all obstructions can be resolved with the urokinase component of human urine. Lu et al. reported that maintaining and opening the PCN tube and regularly flushing it through the opening outside of the body could effectively prevent DJUS blockage [30]. In the present study, irrigation was not routinely performed for cases in which a hematoma was present in the immediate postoperative or follow-up nephrogram. This might have affected the overall long-term DJUS patency results; therefore, further large cohort studies are necessary. 

According to a prospective study by Byun et al., the length and etiology of ureteral stenosis influence the effect of balloon dilatation [31]. The success rate of balloon dilatation was high when the stenosis length was less than 2 cm (short stricture (<2 cm): 69 ± 16%; long stricture (>2 cm): 19 ± 29%), as identified after a meta-analysis [20]. Previous infections, urinary extravasation, and vascular compromise may be the factors involved in the formation of long ureteral strictures and their resistance to dilation [20]. Most studies in this field have shown that balloon dilatation achieves better efficacy for shorter stenoses. In our study, although not statistically significant, the early dysfunction rate varied depending on stricture length, with mild at 0%, moderate at 25%, and severe at 35.7%, indicating that the effectiveness of balloon dilatation decreased as the segmental stricture length increased. Moreover, when the stricture length was long, the long-term DJUS patency was poor, regardless of whether balloon dilatation was performed, in our multivariate Cox proportional analysis. In cases of long ureteral strictures, the two ipsilateral ureteral stents introduced by Liu and Hrebinko in 1998 can be good alternatives [32]. They reported that the main advantages were the increased stiffness of the two stents to reduce kinking and the increased potential space between the stents to preserve the flow passage. Further studies with larger patient cohorts are warranted.

This study had several limitations. First, the male-to-female ratio of the patients including our study was approximately 6.875 to 1. However, the incidence of bladder cancer was 3–4 times higher in men than in women [33]. Future studies should take this into consideration and ensure the necessary adjustments of these variables. Second, the number of patients enrolled was small. Third, it was a retrospective study, which prevented control over certain conditions, including the day of PCN placement, the time between DJUS and PCN insertion, and the day of follow-up. Finally, the median follow-up duration was 88 days in this study. At our institution, DJUSs were replaced or removed after 3–4 months, which limited the duration of follow-up research on their long-term patency. Consequently, this inevitably led to an slight insufficiency in fully assessing the long-term outcomes and potential complications associated with percutaneous stenting.

## 5. Conclusions

Percutaneous insertion of DJUSs is safe and effective in patients with UIAS who have undergone radical cystectomy. The early DJUS patency rate can be improved by decreasing the blood clot retention grade and stricture length, and performing balloon dilatation. Furthermore, in cases in which the blood clot retention grade is high or the ureteral stricture is long, the overall DJUS patency may not be favorable; therefore, close monitoring is necessary.

## Figures and Tables

**Table 1:** Baseline demographics and clinical data of the study patients.

Characteristics	N (%)
Sex (%)	
Male	55 (87.3)
Female	8 (12.7)
Age, years (range)	68.8 ± 6.7
≤65	17 (27.0)
66–75	34 (54.0)
>75	12 (19.0)
Hydronephrosis grade	
Mild	15 (23.8)
Moderate	35 (55.6)
Severe	13 (20.6)
Blood clot retention grade	
1	38 (60.3)
2	18 (28.6)
3	7 (11.1)
Direction of DJUS ^1^	
Right	24 (38.2)
Left	39 (61.8)
Length of ureteral stricture	
Mild (<1 cm)	18 (28.6)
Moderate (1–2 cm)	15 (23.8)
Severe (>2 cm)	30 (47.6)
Size of DJUS	
6F	29 (46.0)
8F	34 (54.0)
Type of DJUS	
Flexima	15 (23.8)
Inlay Optima	48 (76.2)
Balloon dilatation	
Yes	30 (47.6)
No	33 (52.4)
BUN before DJUS	30.48 ± 15.27
Cr before DJUS	2.1 ± 1.4

^1^ DJUS: double-J ureteral stent.

**Table 2:** Multivariable logistic regression analysis of risk factors for early DJUS ^1^ dysfunction in patients who underwent radical cystectomy.

Characteristics	Univariate		Multivariate	
	OR (95% CI)	*p*	OR (95% CI)	*p*
Sex				
Male	2.000 (0.369–10.826)	0.421		
Female				
Age				
≤65	1	0.533		
66–75	0.431 (0.062–3.012)	0.396		
>75	0.333 (0.048–2.328)	0.268		
Hydronephrosis				
Mild	1	0.125		
Moderate	0.600 (0.169–2.130)	0.429		
Severe	2.400 (0.524–10.992)	0.259		
Blood clot retention grade				
1	1	0.004	1	0.019
2	5.893 (1.727–20.106)	0.005	6.922 (1.611–29.744)	0.009
3	9.375 (1.525–57.621)	0.016	5.543 (0.737–41.714)	0.096
Direction of DJUS				
Right	0.960 (0.336–2.739)	0.939		
Left				
Balloon dilatation				
Yes	0.286 (0.097–0.850)	0.024	0.186 (0.046–0.743)	0.017
No				
Length of ureteral stricture				
Mild (<1 cm)	1	0.041	1	0.066
Moderate (1–2 cm)	7.000 (1.173–41.759)	0.033	8.715 (1.164–65.226)	0.035
Severe (>2 cm)	8.000 (1.560–41.033)	0.013	7.646 (1.245–46.953)	0.028
Size of DJUS				
6F	0.752 (0.269–2.098)	0.586		
8F				
Type of DJUS				
Flexima	1.596 (0.493–5.163)	0.435		
Inlay Optima				

^1^ DJUS: double-J ureteral stent.

**Table 3:** Univariate and multivariate Cox’s proportional hazards analysis of risk factors associated with the overall patency of DJUSs ^1^ in patients who had undergone radical cystectomy.

Characteristics	Univariate		Multivariate	
	OR (95% CI)	*p*	OR (95% CI)	*p*
Sex				
Male	1.927 (0.457–8.133)	0.372		
Female				
Age				
≤65	1	0.147		
66–75	0.507 (0.226–1.134)	0.098		
>75	0.390 (0.123–1.233)	0.109		
Hydronephrosis				
Mild	1	0.028	1	0.534
Moderate	0.936 (0.355–2.467)	0.894	1.113 (0.415–2.989)	0.831
Severe	2.869 (1.010–8.149)	0.048	1.796 (0.578–5.578)	0.311
Blood clot retention grade				
1	1	<0.001	1	<0.001
2	6.370 (2.732–14.852)	<0.001	6.044 (2.463–14.831)	<0.001
3	6.248 (1.868–20.902)	0.003	6.330 (1.810–22.140)	0.004
Direction of DJUS				
Right	0.646–1.413	0.820		
Left				
Balloon dilatation				
Yes	0.836 (0.397–1.762)	0.638		
No				
Length of ureteral stricture				
Mild (<1 cm)	1	0.013	1	0.049
Moderate (1–2 cm)	4.386 (1.157–16.626)	0.030	4.081 (1.054–15.798)	0.042
Severe (>2 cm)	6.738 (1.889–24.032)	0.003	5.518 (1.399–21.764)	0.015
Size of DJUS				
6F	0.900 (0.427–1.895)	0.781		
8F				
Type of DJUS				
Flexima	1.783 (0.804–3.953)	0.155		
Inlay Optima				

^1^ DJUS: double-J ureteral stent.

## Data Availability

All data are available through contacting the authors.
